# The mRNA expression and secretion of granzyme B are up-regulated via the histamine H2 receptor in human CD4^+^ T cells

**DOI:** 10.1007/s00011-023-01759-3

**Published:** 2023-07-20

**Authors:** Damilola Dawodu, Sophie Sand, Eirini Nikolouli, Thomas Werfel, Susanne Mommert

**Affiliations:** Department of Dermatology and Allergy, Hannover Medical School, Carl-Neuberg-Str. 1, 30625 Hannover, Germany

**Keywords:** Atopic dermatitis, Th2-polarized CD4^+^ T cells, Histamine, Granzyme B

## Abstract

**Introduction:**

Granzyme B (GZMB), a serine protease with cytotoxic and immunomodulatory functions, shows elevated levels in blood plasma of patients with atopic dermatitis (AD). It has been observed that GZMB expression in CD4^+^ and CD8^+^ T cells is higher in lesional skin in AD than in healthy skin. Since histamine is present in high concentration in the skin of AD patients, we investigated the regulation of GZMB in human CD4^+^ T cells by histamine.

**Methods:**

Naïve CD4^+^ T cells polarized into Th2 cells, total CD4^+^ T cells treated with IL-4 for 72 h and CD4^+^ T cells isolated from healthy donors and AD patients were investigated. The cells were stimulated with histamine or with different histamine-receptor agonists. Gene expression was evaluated by RNA-Seq. GZMB mRNA expression was detected by quantitative real time PCR, whereas GZMB secretion was measured by ELISpot and ELISA. T cell degranulation was evaluated by flow cytometry using CD107a surface expression as a degranulation marker.

**Results:**

By RNA-Seq, we identified the up-regulation of various genes of the cytotoxic pathway, in particular of GZMB, by histamine in Th2-polarized CD4^+^ T cells. In Th2-polarized CD4^+^ T cells and in CD4^+^ T cells activated by IL-4 the mRNA expression of GZMB was significantly up-regulated by histamine and by histamine H2 receptor (H2R) agonists. The induction of GZMB secretion by histamine was significantly higher in CD4^+^ T cells from AD patients than in those from healthy donors. CD107a surface expression was up-regulated by trend in response to histamine in Th2-polarized CD4^+^ T cells.

**Conclusion:**

Our findings may help to elucidate novel mechanisms of the H2R and to achieve a better understanding of the role of GZMB in the pathogenesis of AD.

**Supplementary Information:**

The online version contains supplementary material available at 10.1007/s00011-023-01759-3.

## Introduction

Defence of cytotoxic lymphocytes and NK cells against virally infected or tumour cells is mediated by the release of serine proteases belonging to the family of granzymes. Among five granzymes expressed in humans, granzyme B (GZMB) represents the most intensively studied protease since its discovery by several groups [1, 2]. Beyond cytotoxic CD8^+^ or CD4^+^ T cells and NK cells, GZMB is also expressed in other immune cells such as mast cells, neutrophils, basophils, dendritic cells and macrophages as well as in non-immune cells, for example in keratinocytes. In T lymphocytes and NK cells, GZMB is constitutively expressed at mRNA level and up-regulated at mRNA and protein level upon T cell activation. Induction of GZMB was observed after stimulation with IL-2 or IL-15 in murine CD4^+^ and CD8^+^ T cells [3]. The mannose-6-phosphate receptor is involved targeting GZMB to acidic lytic granules in lymphocytes [4]. The pore-forming protein perforin 1 (PRF1) is also stored in the granules of CD4^+^ and CD8^+^ cytotoxic T lymphocytes and facilitates GZMB internalization into the target cell as well as playing a role for the entry of granzymes from the granule into the cytosol of respective cells. GZMB targets various substrates in the cytosol or nucleus to induce apoptosis via different pathways [5]. However, the exclusivity of the intracellular capacity of GZMB in the context of apoptosis has been recently questioned, since GZMB has been found extracellularly in several tissues and body fluids [6]. One of the suspected sources of the extracellular accumulation of GZMB could be the secretion of GZMB without expression of PRF1 from mast cells [7] or keratinocytes [8]. Also during effector-target cell engagement active GZMB escapes from the immunological synapse into the extracellular milieu [9]. Multiple extracellular roles for GZMB have been described including the proteolytic cleavage of extracellular matrix molecules such as fibronectin, collagen or basement membrane proteins [10]. In terms of these intra- and extracellular functions of GZMB, this protease is considered as a pro-inflammatory mediator, which contributes to the pathogenesis of multiple inflammatory diseases. An emerging role of GZMB due to its expression in macrophages, mast cells [7], T cells and keratinocytes [11] was described in dermatological diseases. In atopic dermatitis (AD), a chronic inflammatory skin disease, characterized by eczematous skin lesions, intense pruritus, epidermal barrier defects, and by a dysregulation of the immune system [12–14], an increase of GZMB expression in CD4^+^ and CD8^+^ T cells was observed. Plasma GZMB levels were detected to be significantly higher in AD patients when compared to healthy controls. Furthermore, the authors found a positive correlation among plasma concentrations of GZMB with the severity score of the disease SCORAD as well as with the severity of pruritus [15]. Since AD is associated with mast cell activation and histamine release, the enhanced concentration of the biogenic amine in the skin may influence the skin infiltrating immune cells. Besides a mixed dermal infiltrate composed of neutrophils, mast cells, macrophages and occasionally of eosinophils a dense infiltrate of activated T cells, which play a prominent role in the pathophysiology of AD, was observed [14, 16, 17]. CD4^+^ T cells and particularly Th2-polarized CD4^+^ T cells express the histamine H1 receptor (H1R), H2R [18] and H4R [19]. In the presence of histamine or of an H4R agonist these T cells express elevated levels of IL-31, a cytokine, which has been identified as a pruritogen in the context of AD [19]. To discover more genes regulated by histamine in CD4^+^ T cells, we applied RNA-Seq as an extensive approach on Th2-polarized CD4^+^ T cells.

By analysing the expression of several genes in Th2-polarized CD4^+^ T cells treated with histamine during the polarization process compared to untreated cells, we identified the up-regulation of various genes of the cytotoxic pathway, in particular of GZMB. The further goal of this study was to confirm the observation of RNA-Seq by analysing GZMB mRNA expression and protein secretion in Th2-polarized CD4^+^ T cells, in CD4^+^ T cells treated with IL-4 and in CD4^+^ T cells from AD patients compared to cells from healthy donors.

## Materials and methods

### Isolation of human naïve or total CD4^+^ T cells

Residual blood samples from platelet (PLT) apheresis disposables used for routine PLT collection of regular anonymous healthy donors (buffy coats) served as source material for the isolation of human peripheral blood mononuclear cells (PBMCs). Venous blood samples were taken from healthy control persons (Caucasian, *n* = 10, 4 females and 6 males mean age 29.3 years) and from patients with moderate-to-severe extrinsic atopic dermatitis (AD) (Caucasian, *n* = 8, 1 female and 7 males mean age 33,25 years). Mean SCORAD was 35.0 ranging from 13.6 to 62.4. Three AD patients received dupilumab as systemic treatment, the others received no systemic treatment. AD patients and healthy control persons were recruited from the Department of Dermatology and Allergy, Hannover Medical School, Hannover, Germany. AD was diagnosed according to the criteria of Hanifin and Rajka [20].

PBMCs were separated by density gradient centrifugation on Pancoll human (PAN-Biotech, Aidenbach, Germany) and afterwards naïve or total CD4^+^ T cells were isolated using respective CD4^+^ T cell Isolation Kits (Miltenyi Biotec Inc., Bergisch Gladbach, Germany).

### Differentiation of naïve CD4^+^ T cells derived from regular anonymous healthy donors (buffy coats) into Th2-polarized CD4^+^ T cells

For the in vitro differentiation of naïve CD4^+^ T cells into Th2 cells, 1.5 × 10^5^ naïve CD4^+^ T cells per well were cultured in 96-well, round bottom plates in modified Iscove’s medium supplemented with 5% v/v human AB serum, nonessential amino acids (1% wt/vol), L-glutamine (1% wt/vol), penicillin/streptomycin (1% wt/vol) and gentamicin (0.5% wt/vol) (Sigma Aldrich; all other media components from Biochrom, Berlin, Germany), at 37 °C in a humidified atmosphere at 5% CO_2_. The cells were stimulated with soluble purified NA/LE Mouse anti-human CD3 (HIT3a; BD Pharmingen, Franklin Lakes, New Jersey, U.S.) and purified NA/LE Mouse anti-human CD28 (CD28.2; BD Pharmingen) antibodies in concentrations 1 μg/mL and 2 μg/mL, respectively. Anti-IFNγ (5 μg/mL; R&D systems), anti-IL12 (500 ng/mL; R&D systems), IL-4 (20 ng/mL; R&D systems) and recombinant human IL-2 (20 ng/mL; R&D systems) were given to the cultures. Th0 CD4^+^ T cells were cultured in the same manner without adding of cytokines. On day 4, the medium of the cells was replaced with fresh medium with (Th2 CD4^+^ T cells) or without (Th0 CD4^+^ T cells) cytokines but for both cell types without stimulating antibodies. On day 7, the cells were split into two wells and fresh medium with or without cytokines was added. On day 8 cells were used for stimulation.

For RNA-Seq, one part of the CD4^+^ T cells was treated with histamine 10 µM during the whole differentiation process and medium changes to Th2-polarized CD^+^ T cells and one part was left non-stimulated.

### Culture of total CD4^+^ T cells derived from regular anonymous healthy donors (buffy coats), from healthy control persons and from patients with moderate-to-severe AD (venous blood)

Total CD4^+^ T cells were cultured in 96 well plate, 1,5 × 10^5^ per well, in presence of 20 ng/mL IL-4 in modified Iscove’s medium supplemented with 4% v/v human AB serum, nonessential amino acids (1% wt/vol), l-glutamine (1% wt/vol), penicillin/streptomycin (1% wt/vol) and gentamicin (0.5% wt/vol), at 37 °C in a humidified atmosphere at 5% CO_2_. After 2 days, the cells were stimulated with histamine or selective agonists for the H1R, H2R and H4R for 6 h and 24 h. After 72 h cultivation total RNA and supernatants were collected.

Since in the following investigations the influence of the in vivo prevailing milieu atopic or healthy was analyzed with regard to the GZMB expression, the duration of the in vitro cultivation was kept short in the next protocol and was not modified by cytokines.

Accordingly, total CD4^+^ T cells derived from healthy control persons and from patients with moderate-to-severe AD were directly cultured in modified Iscove’s medium and stimulated immediately with histamine or selective agonists for the H2R and H4R for 6 h or 24 h.

### RNA sequencing

#### Library generation, quality control, and quantification

50 ng of total RNA per sample, histamine treated Th2-polarized CD4^+^ T cells versus non-treated Th2-polarized CD4^+^ T cells from four different donors, were utilized as input for mRNA enrichment procedure with ‘NEBNext^®^ Poly(A) mRNA Magnetic Isolation Module’ (E7490L; New England Biolabs) followed by stranded cDNA library generation using ‘NEBNext^®^ Ultra II Directional RNA Library Prep Kit for Illumina’ (E7760L; New England Biolabs). All steps were performed as recommended in user manualE7760 (Version 1.0_02-2017; NEB) except that all reactions were downscaled to 2/3 of initial volumes. Furthermore, one additional purification step was introduced at the end of the standard procedure, using 1x ‘Agencourt^®^ AMPure^®^ XP Beads’ (#A63881; Beckman Coulter, Inc.).cDNA libraries were barcoded by single indexing approach, using ‘NEBNext Multiplex Oligos for Illumina–Set 1’ (Index Primer 1-12). All generated cDNA libraries were amplified by 11 cycles of final pcr.

Fragment length distribution of individual libraries was monitored using ‘Bioanalyzer High Sensitivity DNA Assay’ (5067-4626; Agilent Technologies). Quantification of libraries was performed by use of the ‘Qubit^®^ dsDNA HS Assay Kit’ (Q32854; ThermoFisher, Braunschweig, Germany).

#### Library denaturation and sequencing run

Equal molar amounts of 12 individually barcoded libraries were pooled. Accordingly, each analyzed library constitutes 8.3% of overall flow cell capacity. The library pool was denatured with NaOH and was finally diluted to 1.5 pM according to the Denature and Dilute Libraries Guide (Document # 15048776 v02; Illumina). 1.3 ml of denatured pool was loaded on an Illumina NextSeq 550 sequencer using a High Output Flowcell for paired-end reads (20024907; Illumina). Sequencing was performed with the following settings: sequence reads 1 and 2: 81 bp each; index read 1: 6 bp.

#### BCL to FASTQ conversion

BCL files were converted to FASTQ files using bcl2fastq Conversion Software version v2.20.0.422 (Illumina).

#### Raw data processing and quality control

Raw data processing was conducted by use of nfcore/rnaseq (version 1.5dev) which is a bioinformatics best-practice analysis pipeline used for RNA sequencing data at the National Genomics Infrastructure at SciLifeLab Stockholm, Sweden. The pipeline uses Nextflow, a bioinformatics workflow tool. It pre-processes raw data from FastQ inputs, aligns the reads and performs extensive quality-control on the results. The genome reference and annotation data were taken from GENCODE.org (Homo sapiens; GRCh38.p12; release 28).

### Histamine receptor ligands

The following histamine receptor ligands were used in this study: Histamine (ALK-Abello, Madrid, Spain) as agonist for all histamine receptors; 2-pyridylethylamine (Tocris Bioscience, Bristol, UK) as selective H1R agonist; amthamine (Tocris Bioscience, Bristol, UK) as selective H2R agonist; ST-1006 (Institute of Pharmaceutical and Medicinal Chemistry, Heinrich Heine University, Duesseldorf, Germany) as H4R agonist [21]; ranitidine (Sigma Aldrich, Deisenhofen, Germany) as selective H2R antagonist. JNJ7777120 (Sigma Aldrich, Deisenhofen, Germany) as selective H4R antagonist. All histamine receptor ligands were used at a concentration of 10 µM. In extensive previous studies we could show that the concentration of 10 µM is optimal to demonstrate and reproduce robust histamine receptor ligand mediated effects [22].

### Quantitative real-time-PCR (q-PCR)

Total RNA was isolated using the ReliaPrep™ RNA Miniprep System (Promega, Walldorf, Germany) or the Micro RNA innuPrep (Analytik Jena, Jena Germany) according to the manufacturer’s instructions. The cDNA was synthesized by reverse transcription QuantiTect reverse transcription kit (Qiagen, Hilden, Germany) or RevertAid First Strand cDNA Synthesis Kit (ThermoFisher).q-PCR was performed according to the MIQE guidelines [23] with Quantitect^®^ primer assays for GZMB (QT01001875), GZMA (QT00015575), PRF1 (QT01869602), rps 20 (ribosomal protein S20) (QT00079247) with primers from Tip Mol Biol (Berlin, Germany) for H2R (F: 5′-TACCAgCTgTCCTgCAAgTg, R. 5′-CCCCAggTggATAgACAgAA) and H4R (F:5′-TgCTAggAAATgCTTTggTC, R:5′-gCgTgTgAgggATgTACAAA) using SYBR^®^ Green according to the manufacturer’s instructions (Qiagen, Hilden, Germany) using the LightCycler 480. The amount of the target mRNA relative to the amount of the reference gene mRNA, ribosomal protein S20 (rps 20), in the same sample was calculated using the comparative Ct method also known as the [delta] Ct method provided by the Software LC 480 (Roche Molecular Biochemicals, Mannheim, Germany).

### Enzyme-linked-immuno-sorbent-assay (ELISA)

5 × 10^5^ fully differentiated Th2-polarized CD4^+^ T cells per well were stimulated with histamine or amthamine (10 µM) for 24 h or left non-stimulated. Cell-free supernatants were taken. The concentration of the serine protease GZMB was analyzed using a commercially available ELISA. The ELISA was performed according to the manufacturer`s instructions (Mabtech, Stockholm, Sweden).

### Enzyme-linked-immunospot assay (ELISpot Assay)

To measure the frequency of GZMB secreting cells at the single cell level, we applied the ELISpot assay (Mabtech). 1 × 10^4^ Th2-polarized CD4^+^ T cells were cultured on a surface (PVDF membrane) coated with a capture antibody directed against GZMB. The cells were cultured in the presence of histamine or amthamine (10 µM) or left non-stimulated. After 24 h, the cells were removed and the secreted GZMB on the membrane was detected by ELISpot assay according to manufacturer´s instructions. The spot forming cells (SFC) were detected using an Elispot reader.

### Detection of lysosomal associated membrane glycoproteins (LAMPs) on Th2-polarized CD4^+^ T cells by flow cytometry

Target cell killing by cytotoxic cells is mediated via perforin-granzyme release toward the immunological synapse. Pre-formed lytic granules, when delivered to the site of secretion, fuse with the plasma membrane. Since the granule core is surrounded by a lipid bilayer consisting of LAMPs, we identified the degranulated cells by measuring the expression of the LAMP protein CD107a by flow cytometry [24]. For this purpose we incubated the Th2-polarized CD4^+^ T cells with a fluorescent dye labelled antibody against CD107a during the stimulation time with histamine at 37 °C and 5% CO_2_ for 6 h. On degranulated cells CD107a is exposed to the cell surface and comes in contact with the antibody only for a short period of time because the CD107a and other lysosomal components are internalized very quickly. Monensin (2 µM, ThermoFisher) was also added to the cells to prevent degradation of the fluorescent dye labelled antibody [25]. After 6 h, the cells were washed with PBS/BSA and expression of CD107a was analysed by flow-cytometry.

### Analysis of chemoattractant receptor-homologous molecule expressed on Th2 cells (CRTH2) expression as a marker for Th2-polarized CD4^+^ T cells by flow cytometry

Th2-polarized CD4^+^ T cells were platted in a density of 1 × 10^5^ cells per well. Fc receptors were blocked by incubation in a buffer containing 10 µg/mL heat-aggravated human immunoglobulin G (IgG; Sigma, Deisenhofen, Germany). An extracellular epitope of CRTH2 was stained with anti-human CRTH2-PE (mouse monoclonal IgG2a, 1 µg/100 µL; Thermo Fisher) the respective isotype control IgG2a was tested in parallel. CD4^+^ T cells were stained with anti-human CD4-APC (1 µg/100 µL; Beckman Coulter, Krefeld, Germany), the respective isotype control IgG1 was tested in parallel. To assess cell viability, Th2-polarized CD4^+^ T cells were incubated with Fixable Viability Dye eFluor^®^ 660 (BD Bioscience) for 30 min before staining.

After washing with PBS/BSA 1%, sample acquisition was performed by flow cytometry (FACS Calibur, Becton Dickinson, Heidelberg, Germany) and the percentage of gated cells was calculated using the Flow Jow Pro software (Becton Dickinson).

### Statistics

For statistical analyses, the software GraphPad Prism Version 8.0 was used (GraphPad software, San Diego, CA, USA). First, we performed methods to test the normal Gaussian distribution of the data. In all our experiments, due to the individual variations of the data the normality tests failed. The non-parametric tests Wilcoxon matched-pairs signed rank test or Friedman Dunn’s multiple comparison test was used and the median is shown in the graph. A *p* value < 0.05 was regarded as statistically significant (*p* < 0.05 was labelled with *, *p* < 0.01 was labelled with **, *p* < 0.001 was labelled with ***, *p* < 0.0001 was labelled with ****).

## Results

### Histamine differentially regulates gene expression profile of Th2-polarized CD4^+^ T cells

**Fig. 1:**
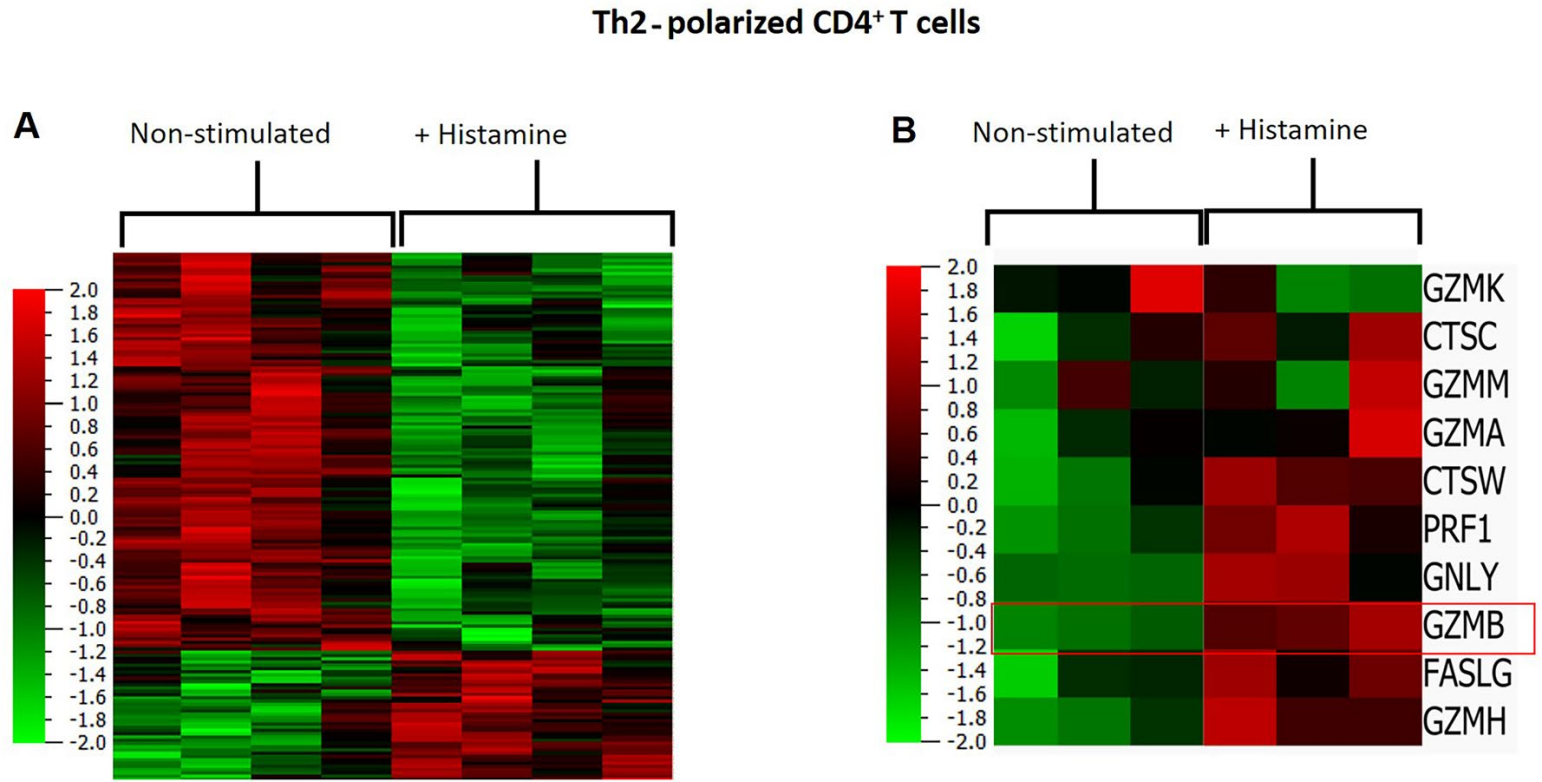
Differentially expressed genes in Th2-polarized CD4^+^ T cells treated with or without histamine. Graphical analysis was done using Qlucore Omics Explorer, (Lund, Sweden). **A** Heatmap showing differential gene expression in untreated Th2-polarized CD4^+^ T cells (left) and Th2-polarized CD4^+^ T cells treated with histamine (right), isolated from four different donors. **B** Heatmap showing differential gene expression of several proteases including granzyme B (GZMB) in untreated Th2-polarized CD4^+^ T cells (left) and Th2-polarized CD4^+^ T cells treated with histamine (Hist; 10 µM) (right), isolated from 3 different donors. The genes that were up-regulated are shown in red. The green areas indicate down-regulated genes

To identify genes that are differentially regulated by histamine in Th2-polarized CD4^+^ T cells, naïve CD4 ^+^ T cells from four healthy donors were differentiated to Th2-polarized CD4^+^ T cells for 11 days with or without 10 µM histamine from day 0 of the differentiation procedure. Total RNA was extracted at day 11 and analyzed by RNA-Seq. First, we aligned each RNA-Seq sample against the human reference genome. A total of 58,381 genes were detected for the reads aligned to the human genome. Differentially expressed genes (DEGs) between histamine treated Th2-polarized CD4^+^ T cells and untreated cells were defined as genes with FDR-adjusted *p* values < 0.05. A total of 568 genes were differentially regulated by histamine; 334 genes were up-regulated while 234 genes were down-regulated (Fig. 1A). Expression of cytotoxic molecule transcripts in Th2-polarized CD4^+^ T cells treated with histamine versus non-treated cells from three out of four different donors is depicted in Fig. 1B. The mRNA of the serine protease GZMB stands out with a clear up-regulation by histamine.

### Granzyme B (GZMB) mRNA expression is up-regulated by histamine and by stimulating the H2R and H4R with specific agonists in Th2-polarized CD4^+^ T cells

**Fig. 2:**
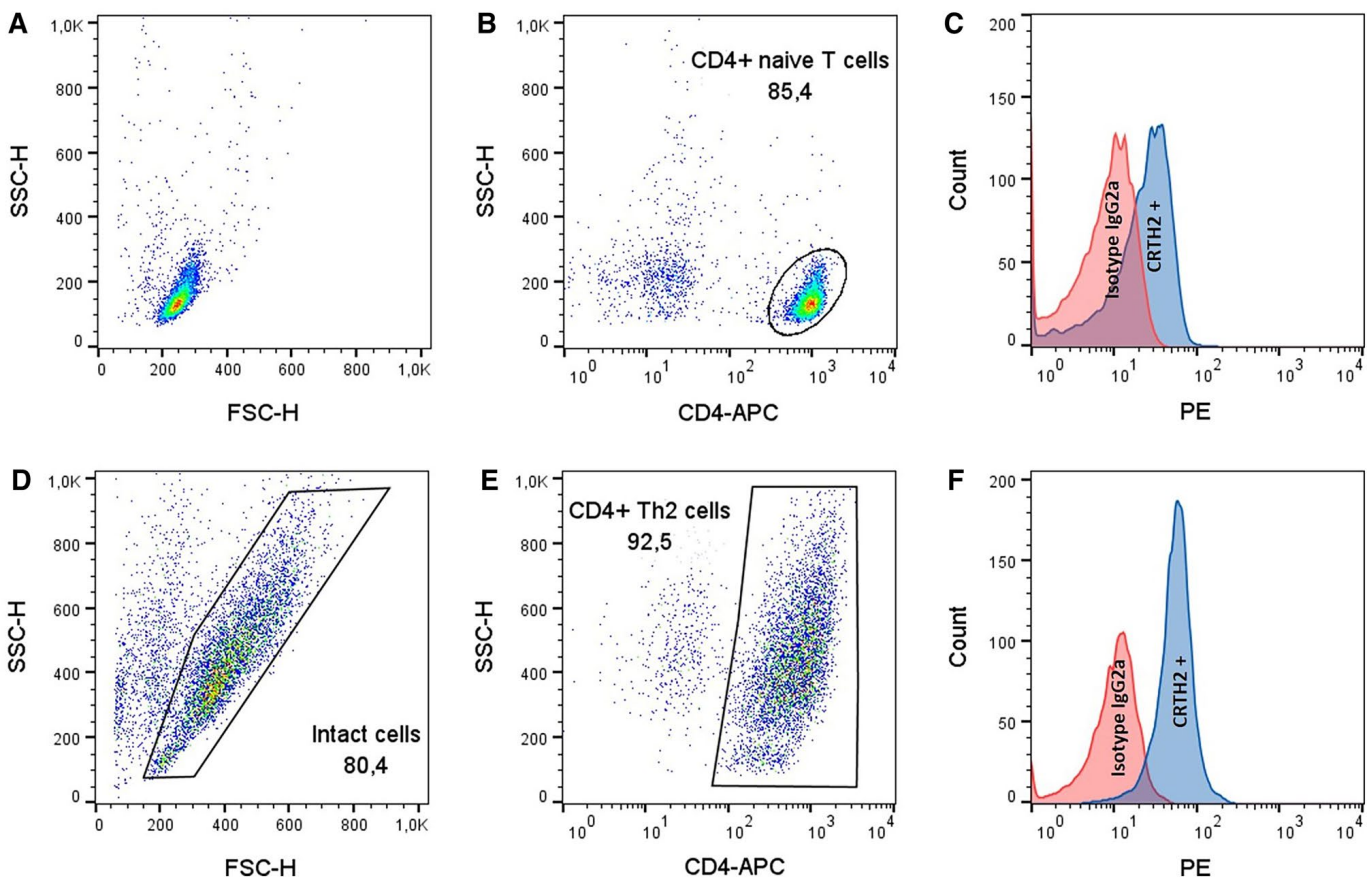
Analysis of expression levels of the chemoattractant receptor-homologous molecule expressed on Th2 cells (CRTH2) on CD4^+^ T cells as marker of Th2 polarization. Human peripheral blood mononuclear cells (PBMCs) were isolated from regular anonymous healthy donors followed by separation of naïve CD4^+^ T cells by magnetic cell sorting and polarized to Th2 cells for eight days. On day 0 and 7 the cells were fluorescently stained with anti-CRTH2-phycoerythrin (PE) and anti-CD4-Allophycocyanin (APC) antibodies with subsequent flow cytometric measurement. **A**–**C** Measurement on day 0. **D**–**F** Measurement on day 7. **A**, **D** Forward-Scatter (FSC) and Side-Scatter (SSC). The dead cells and debris were excluded from further analysis due to their small size by setting a gate in the FSC scatter. The percentage at the bottom right describes the proportion of the remaining, intact cells in **D**. **B** and **E** CD4^+^ T cells were gated. **C**, **F** Histogram overlay: isotype IgG2a versus CRTH2 expression. Representative example out of 6 (day 0) and 7 (day 7) experiments

To confirm the mRNA up-regulation of GZMB which was detected by RNA-Seq also by q-PCR, we isolated peripheral blood mononuclear cells (PBMCs) from regular anonymous healthy donors (buffy coats) followed by separation of naïve CD4^+^ T cells by magnetic cell sorting. Purity of naïve CD4^+^ T cells is depicted in Supplementary Fig. 1. Naïve CD4^+^ T cells were cultured under Th0 or Th2 polarizing conditions for 8 days. Expression of chemoattractant receptor-homologous molecule expressed on Th2 cells (CRTH2), which binds to prostaglandin D2, has proven to point to polarization into Th2-polarized CD4^+^ T cells [26]. We detected by flow cytometry that the expression of the Th2 marker CRTH2 increases during the polarization of naïve CD4^+^ T cells to Th2-polarized CD4^+^ T cells (Fig. 2).

**Fig. 3:**
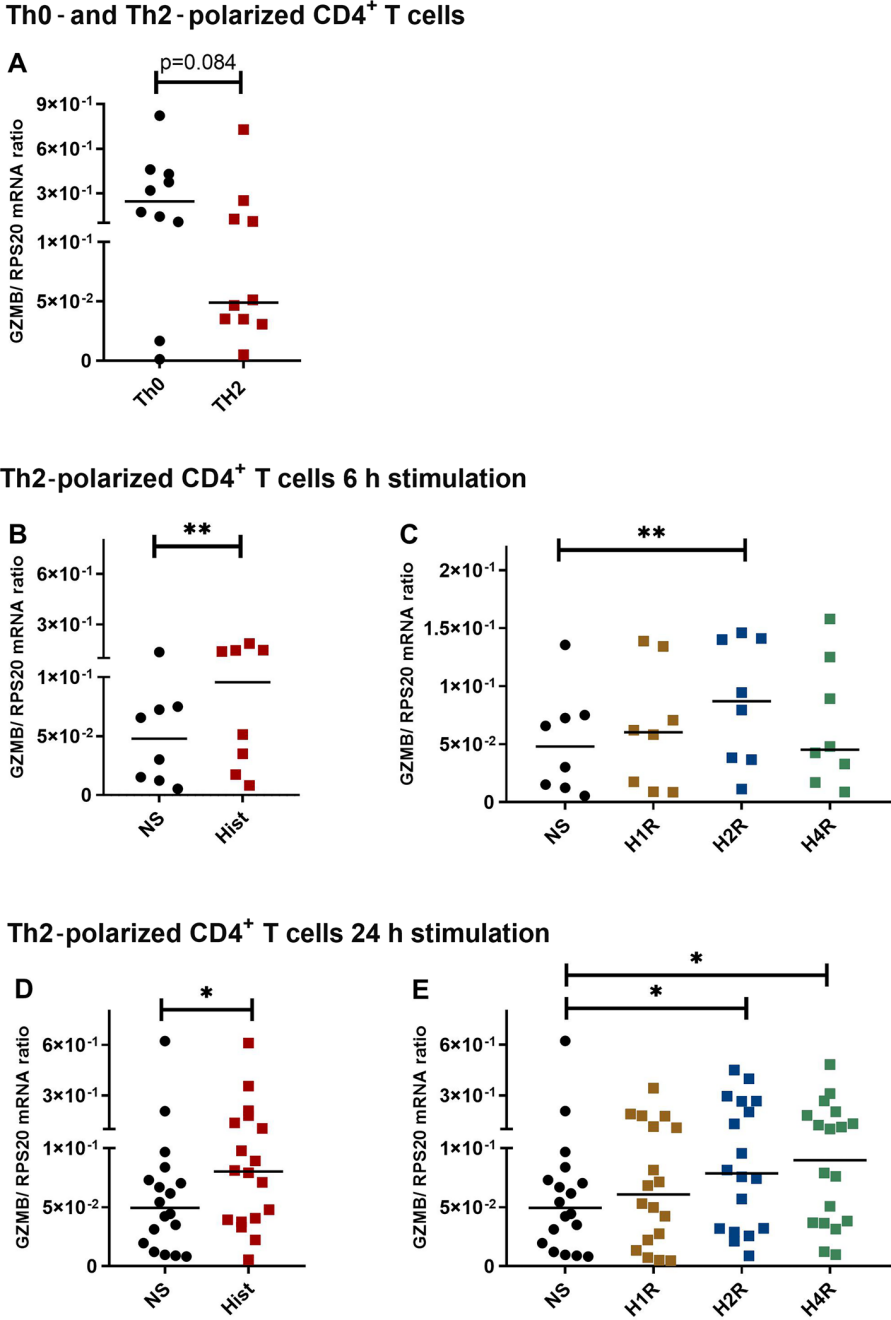
Granzyme B (GZMB) mRNA expression is up-regulated by histamine and by stimulating the H2R and H4R with specific agonists in Th2-polarized CD4^+^ T cells. CD4^+^ T cells were polarized to Th0 and Th2 cells for eight days. **A** Constitutive GZMB mRNA expression was measured in Th0-polarized CD4^+^ T versus Th2-polarized CD4^+^ T cells. Th2-polarized CD4^+^ T cells were stimulated with histamine (Hist), the histamine H1 receptor agonist 2-pyridylethylamine (H1R), the H2R agonist amthamine (H2R) or the H4R agonist ST-1006 (H4R) (each ligand 10 µM) **B**, **C** for 6 h; **D, E** for 24 h or left non-stimulated (NS). GZMB mRNA expression was detected by q-PCR. GZMB mRNA expression relative to the rps 20 mRNA expression (reference gene) is shown as target/reference (GZMB/RPS20) ratio. Data are shown as individual values with medians. Significant differences, as determined by the Wilcoxon matched-pairs signed-rank test in **A**, **B** and **D**, or by the Friedman Dunn’s multiple comparison test in **C** and **E** are indicated as follows: *P < 0.05; **P < 0.01; **A** (*n* = 10 independent donors and experiments); **B**, **C** (*n* = 8 independent donors and experiments); **D**, **E** (*n* = 18 independent donors and experiments)

We detected high constitutive GZMB expression in CD3/CD28 activated Th0-polarized CD4^+^ T and in Th2-polarized CD4^+^ T cells. However, Th2 cells, which were stimulated with IL-4 during the polarization period, showed lower constitutive GZMB expression by trend in comparison to Th0-polarized CD4^+^ T cells (Fig. 3A).

To confirm our results obtained by RNA-Seq, we investigated the regulation of GZMB mRNA expression in response to histamine and different histamine receptor agonists in human Th2-polarized CD4^+^ T cells. We stimulated the cells with histamine and agonists selective for the H1R, H2R and H4R. We detected an up-regulation of GZMB mRNA expression upon histamine after 6 h (median twofold induction) and after 24 h stimulation (Fig. 3B, D) which was mainly mediated via the H2R after 6 h (median 1.82-fold induction) and mediated via H2R and H4R after 24 h stimulation (Fig. 3C, E).

We performed blocking experiments by incubating the Th2-polarized CD4^+^ T cells for 30 min with the H2R antagonist ranitidine or with the H4R antagonist JNJ7777120 before stimulation with the H2R agonist amthamine or with the H4R agonist ST-1006 respectively for 24 h.

In four of five experiments we observed a blocking effect of the H2R antagonist ranitidine, whereas we observed only in one of these experiments a blocking effect of the H4R antagonist JNJ7777120 (Supplementary Fig. 3).

### Granzyme A (GZMA) and perforin 1 (PRF1) mRNA expressions are not regulated by histamine in Th2-polarized CD4^+^ T cells

**Fig. 4:**
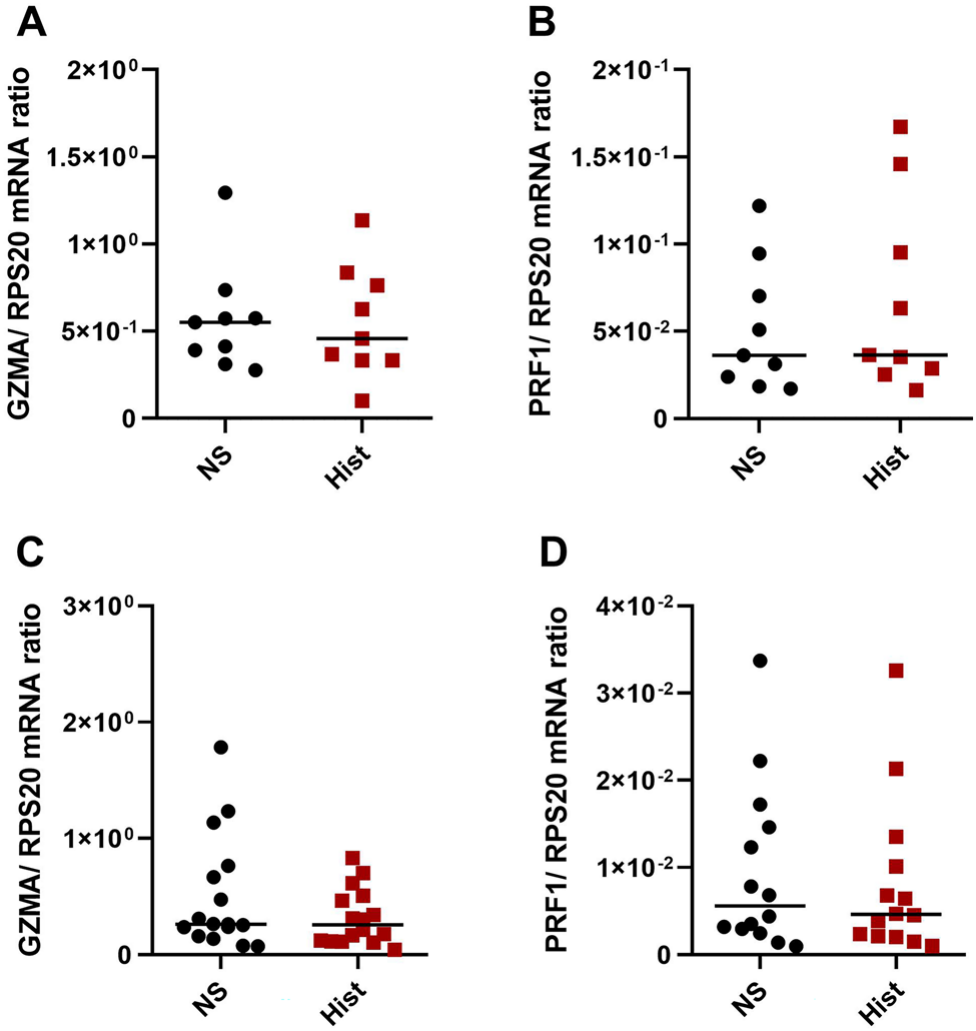
Granzyme A (GZMA) and Perforin 1 (PRF1) mRNA expression are not regulated by histamine in Th2-polarized CD4^+^ T cells. Th2-polarized CD4^+^ T cells cultured for 8 days were stimulated with histamine (Hist; 10 µM) for 6 h (**A**, **B**) and for 24 h (**C**, **D**) or left non-stimulated (NS). **A**, **C** GZMA and **B**, **D** PRF1 mRNA expressions were detected by q-PCR. GZMA mRNA or PRF1 mRNA expression relative to the rps 20 mRNA expression (reference gene) is shown as target/reference (GZMA/RPS 20 or PRF1/RPS 20) ratio. Data are shown as individual values with medians. **A** and **B** (*n* = 9 independent donors and experiments). **C** (*n* = 16 independent donors and experiments) and **D** (*n* = 14 independent donors and experiments)

We investigated if other lytic effector molecules, which are central to cytotoxic functions of T cells are also regulated by histamine. Therefore, we analysed GZMA (Fig. 4A) and PRF1 (Fig. 4B) mRNA expression in Th2-polarized CD4^+^ T cells stimulated with histamine for 6 h and for 24 h (Fig. 4C, D). We could not detect a reliable regulation of the mRNA expression of the lytic effector molecules GZMA and PRF1 in response to histamine.

### Higher H2R mRNA expression when compared to the H4R mRNA expression in Th2-polarized CD4^+^ T cells

By stimulating the H2R on Th2-polarized CD4^+^ T cells, we detected a significant up-regulation of GZMB mRNA expression. With this in mind, we analysed the mRNA expression levels of the H2R and H4R in these cells during the differentiation process of CD4^+^ T cells to Th2-polarized CD4^+^ T cells and in fully differentiated Th2-polarized CD4^+^ T cells after 8 days.

**Fig. 5:**
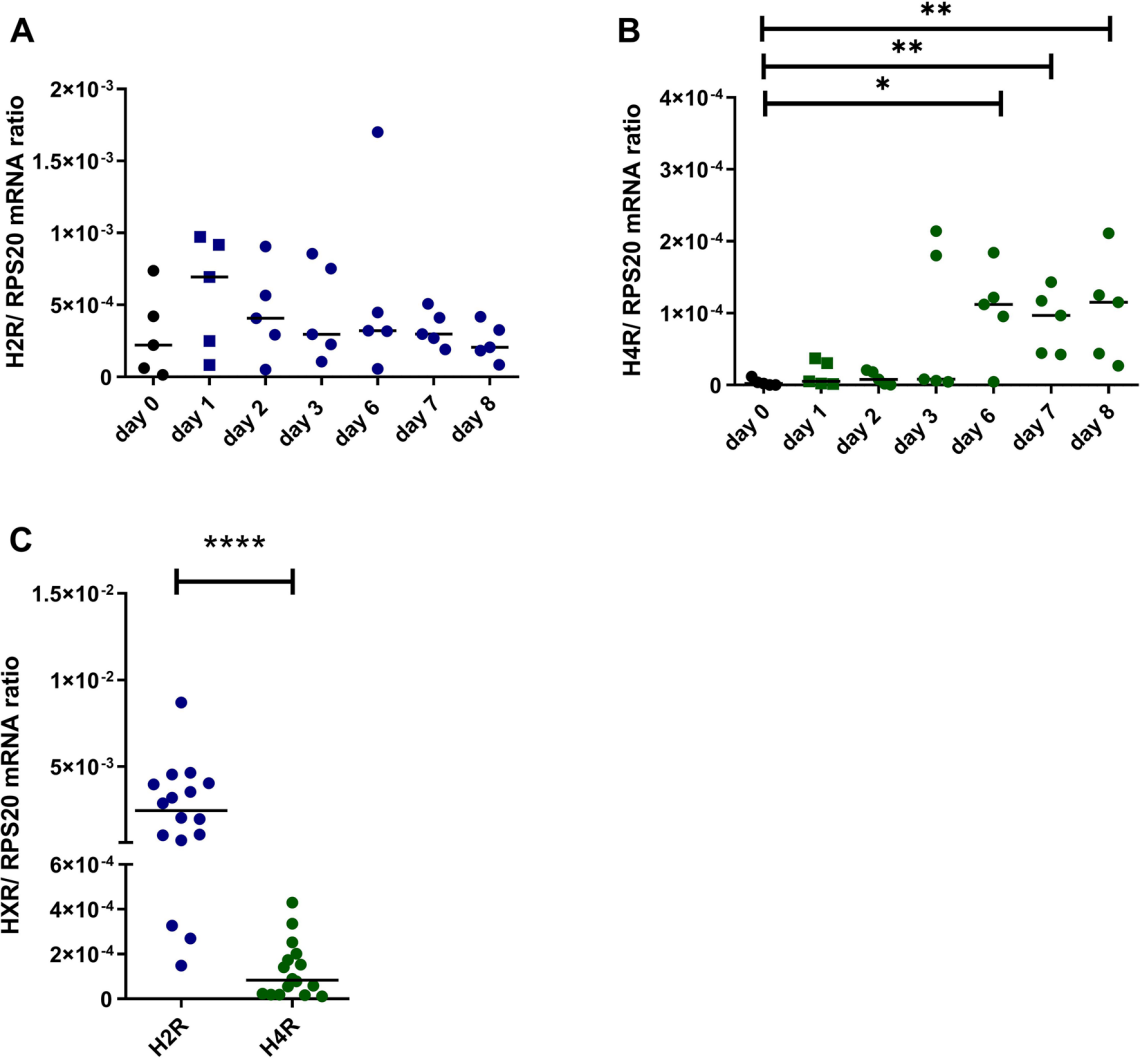
Higher H2R mRNA expression when compared to the H4R mRNA expression in Th2-polarized CD4^+^ T cells. CD4^+^ T cells were differentiated to Th2-polarized CD4^+^ T cells, mRNA expression levels **A** of the H2R and **B** of the H4R were measured at different time points as indicated. **C** Th2-polarized CD4^+^ T cells were cultured for 8 days. H2R and H4R mRNA expressions were detected by q-PCR at day 8. HXR mRNA expression relative to the rps 20 mRNA expression (reference gene) is shown as target/reference (HXR/RPS20) ratio. Data are shown as individual values with medians. Significant differences, as determined by the Wilcoxon matched-pairs signed-rank test in C, or by the Friedman Dunn´s multiple comparison test in B are indicated as follows: *P< 0.05; **P<0.01; ****P < 0.0001; **A** and **B** (*n* = 5 independent donors and experiments); **C** (*n* = 16 independent donors and experiments)

Interestingly, we observed a slight up-regulation of the H2R mRNA expression at day 1 which could be attributed to the activation of the cells by adherence or by the new medium containing M-CSF. However, there were no evident differences of the H2R mRNA expression levels during the differentiation process at day 1 until day 8. In contrast, the H4R mRNA expression was slightly up-regulated at day 6 and this level remained stable until day 8 (Fig. 5A, B).

Comparing the H2R/RPS 20 mRNA ratio versus H4R/RPS 20 mRNA ratio at day 8, we recognized a significantly higher mRNA expression of the H2R when compared to the H4R in Th2-polarized CD4^+^ T cells (Fig. 5C).

Of note, the expression levels of the H2R or H4R mRNA were strongly dependent on the different donors and varied between the individuals.

### Granzyme B (GZMB) protein production is significantly increased after stimulation with histamine for 24 h in Th2-polarized CD4^+^ T cells

**Fig. 6:**
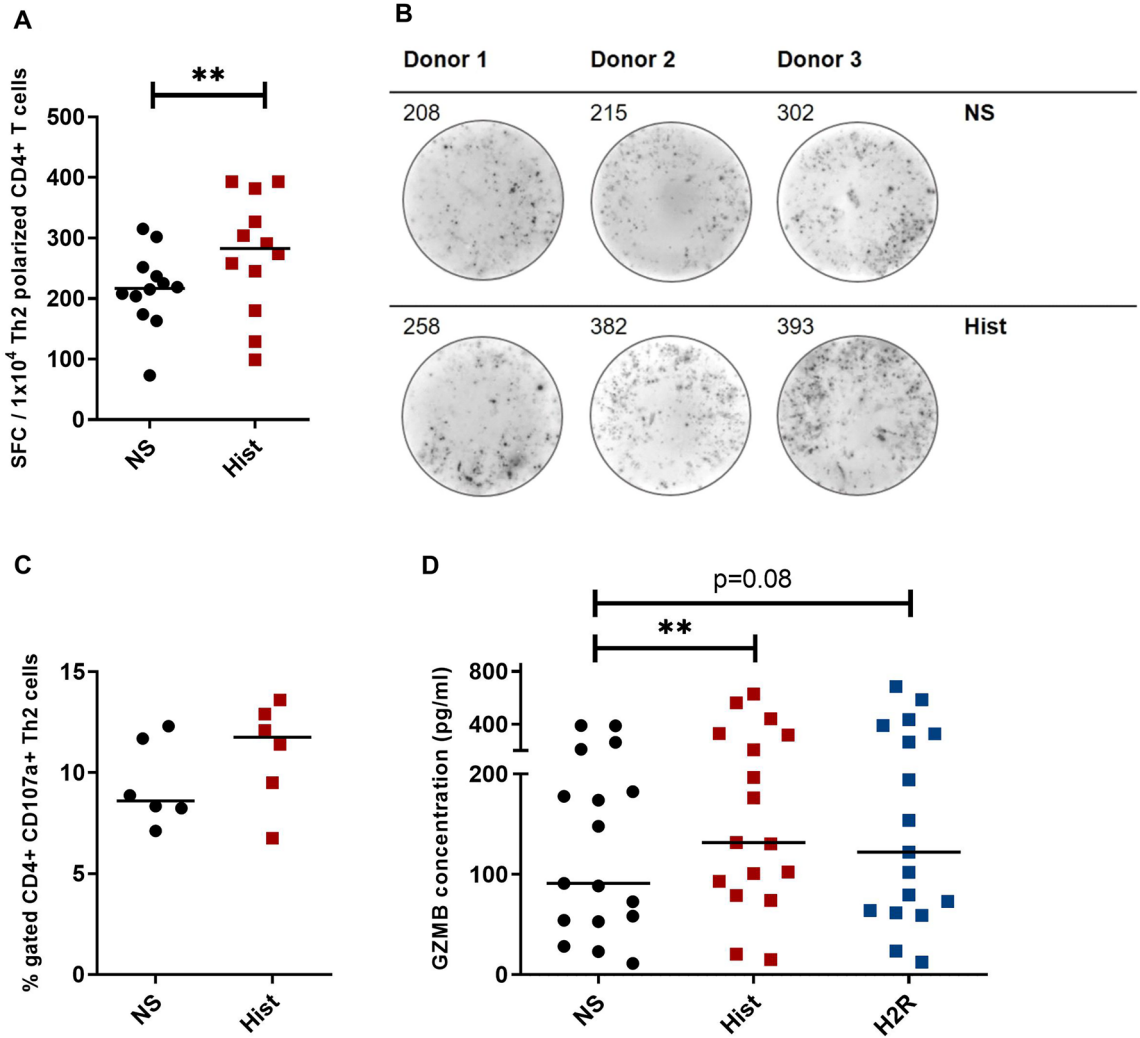
Granzyme B (GZMB) secretion is significantly increased after stimulation with histamine or with the H2R agonist amthamine in Th2-polarized CD4^+^ T cells. Th2-polarized CD4^+^ T cells cultured for 8 days were stimulated or left non-stimulated (NS) with histamine (10 µM) for 24 h. During the stimulation time, the cells were incubated on a PVDF membrane coated with an anti-GZMB antibody (1 × 10^4^ cells/membrane). Spot-forming cells (SFCs), which represent GZMB-secreting cells, were counted on the membrane using an ELISpot reader. **A** The number of SFCs per 1 × 10^4^ Th2-polarized CD4^+^ T cells for non-stimulated (NS) and histamine (Hist) stimulated cells is depicted. **B** Three different membranes from representative donors showing the characteristic SFCs are presented. The number of counted SFCs is displayed at the upper left. **C** Th2-polarized CD4^+^ T cells cultured for eight days were stimulated or left non-stimulated (NS) with histamine (10 µM) for 6 h. CD107a surface expression was analysed by flow cytometry. Percentage of CD107a^+^/CD4^+^ polarized Th2 cells in the upper right quadrant of the dot blots from different donors is depicted in the graph. **D** Th2-polarized CD4^+^ T cells cultured for 8 days were stimulated or left non-stimulated (NS) with histamine (Hist; 10 µM) or with the H2R agonist amthamine (H2R; 10 µM) for 24 h. The GZMB protein concentration in the supernatants (Dilution 1:100) was measured by ELISA. Data are shown as individual values with medians. Significant differences, as determined by the Wilcoxon matched-pairs signed-rank test in **A** or by the Friedman Dunn’s multiple comparison test in **D** are indicated as follows: **P < 0.01; A (*n* = 12 independent donors and experiments); **C** (*n* = 6 independent donors and experiments) and D (*n* = 17 independent donors and experiments)

To analyse the influence of histamine on GZMB expression at protein level, we performed an ELISpot assay using a polyvinylidene fluoride membrane 96-well plate pre-coated with an antibody specific to the secreted GZMB. Th2-polarized CD4^+^ T cells were added to the plate and attached to the coated membrane. Cells were stimulated and the secreted protein was fixed to the anti-GZMB antibody and depicted as a spot-forming cell (SFC). We detected a significantly higher amount of SFCs, which represent GZMB-secreting cells, in the histamine-stimulated samples when compared to the non-stimulated cells (Fig. 6A, B).

The serine protease GZMB is known to be stored in cytotoxic granules of lymphocytes. The secretory release from these granules or lysosomes is accompanied by cell surface expression of transmembrane proteins that usually, in inactive situations, are localized in membranes of secretory lysosomes. We investigated the expression of the lysosomal-associated trans membrane protein CD107a on Th2-polarized CD4^+^ T cells after histamine stimulation for 6 h by flow cytometry. Histamine showed a 1.4-fold increase of the percentage of CD107a^+^ cells whereas stimulation with SEB (data not shown), known as positive control, caused a 3.18-fold increase of the percentage of CD107a^+^ cells when compared to the non-stimulated cells (Fig. 6C).

These data were confirmed by the results of a GZMB ELISA. GZMB protein production was up-regulated in the supernatants of Th2-polarized CD4^+^ T cells stimulated for 24 h with histamine (median 1.45-fold induction) and in cells stimulated with the H2R agonist amthamine (median 1.35-fold induction) when compared to non-stimulated cells (Fig. 6 D).

### Granzyme B (GZMB) mRNA expression is up-regulated by histamine and by stimulating the H2R in IL-4 treated CD4^+^ T cells

**Fig. 7:**
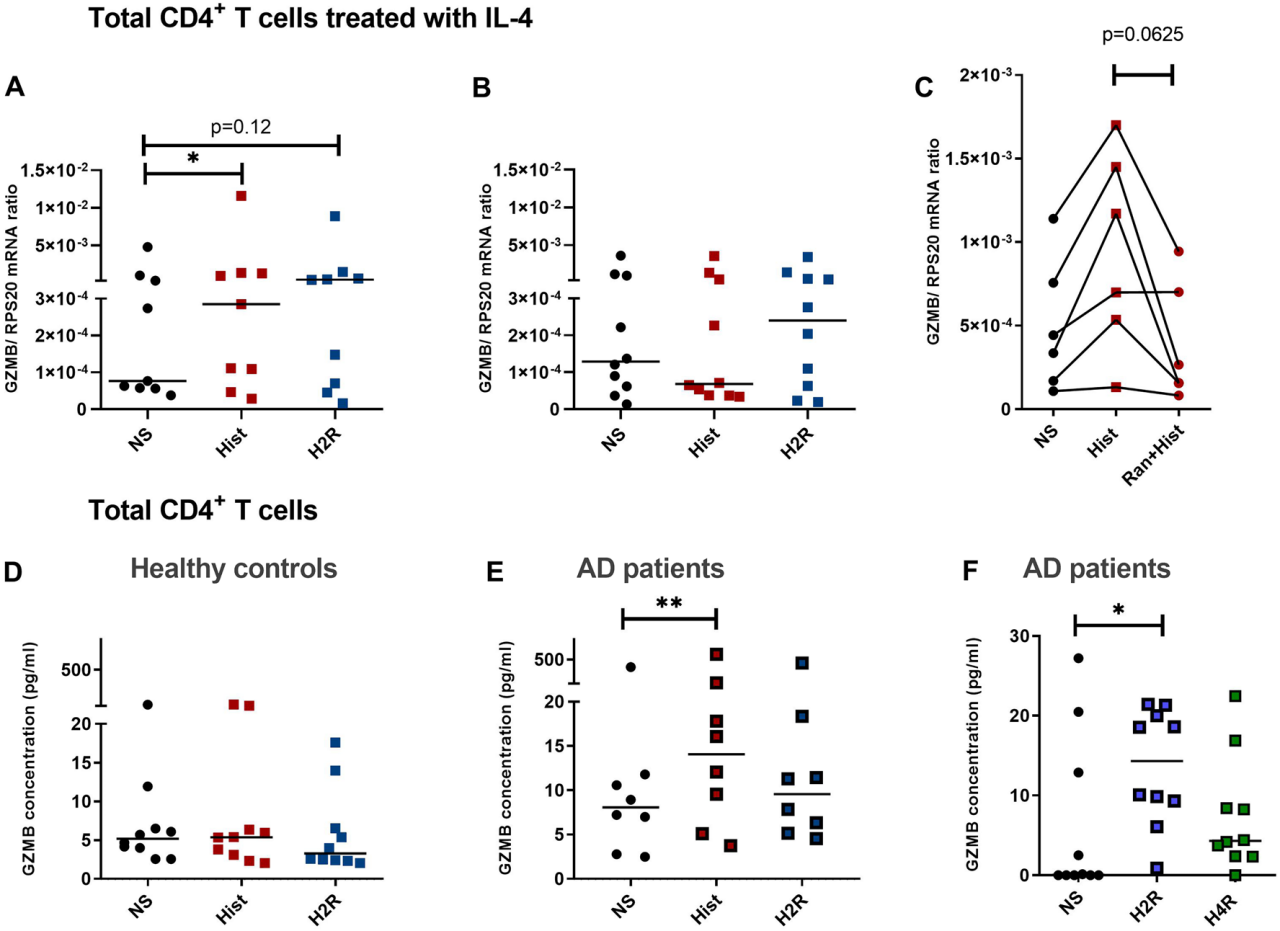
Granzyme B (GZMB) mRNA expression is up-regulated by histamine and by stimulating the H2R in CD4^+^ T cells treated with IL-4. GZMB secretion is up-regulated in CD4^+^ T cells from atopic dermatitis (AD) patients. Total CD4^+^ T cells obtained from buffy coats were cultured in the presence of IL-4 for 72 h. GZMB expression was detected by q-PCR. GZMB mRNA expression relative to the rps 20 mRNA expression (reference gene) is shown as target/reference (GZMB/RPS20) ratio (**A**–**C**). GZMB secretion was measured by ELISA in supernatants of CD4^+^ T cells (**D**) from healthy control persons and **E**, **F** from AD patients after 24 h stimulation. The cells were stimulated or left non-stimulated (NS) with histamine (Hist; 10 µM), with the H2R agonist amthamine (H2R; 10 µM) or with the H4R agonist ST-1006 in (H4R; 10 µM). **A** For 6 h and **B** for 24 h, **C** for blocking experiments the cells were pre-incubated for 30 min with the H2R antagonist ranitidine (Ran + Hist; each 10 µM) before stimulation with histamine for 6 h. Data are shown as individual values with medians. Significant differences, as determined by the Wilcoxon matched-pairs signed-rank test in **C** or by the Friedman Dunn’s multiple comparison in **A**, **E** and **F** are indicated as follows: *P < 0.05; **P < 0.01; **A** (*n* = 9 independent donors and experiments); **B**, **D**, **F** (*n* = 10 independent donors and experiments); **C** (*n* = 6 independent donors and experiments); **E** (*n* = 8 independent donors and experiments)

In vitro differentiation of naïve T cells into Th2 effector cells is a highly regulated process. T cells may deviate from their in vivo counterparts. Therefore, we investigated the histamine effect also in a fraction of total CD4^+^ T cells, which were treated with IL-4 for 72 h only but without T cell and costimulatory receptor stimulations. Because of the lack of these activations, the CD4^+^ T cells showed a considerably lower constitutive GZMB mRNA expression when compared to Th2-polarized CD4^+^ T cells. After 72 h CD4^+^ T cells were stimulated with histamine and the H2R selective agonist amthamine. In accordance to our observations in Th2-polarized CD4^+^ T cells, we detected an up-regulation of GZMB mRNA expression by histamine (median 3.72-fold induction) and by the H2R agonist amthamine by trend (median 5.35-fold induction) after a 6 h stimulation period (Fig. 7A) but not significant after a 24 h stimulation period (Fig. 7B). The histamine-induced up-regulation of GZMB mRNA was partly blocked by pre-incubation with the selective H2R antagonist ranitidine (Fig. 7C).

### GZMB protein secretion is up-regulated by histamine and by stimulating the H2R in CD4^+^ T cells obtained from atopic dermatitis (AD) patients when compared to cells from healthy controls

It was published that plasma levels of GZMB positively correlate with the severity score SCORAD of AD, with serum levels of CCL17 (a marker of the severity of AD) or with the levels of itch related molecules [15]. GZMB had also been observed to contribute to AD severity through cleavage of adhesion molecules, which are important in maintaining an intact skin barrier function [10]. We investigated total CD4^+^ T cells obtained from patients with moderate-to-severe AD in comparison to CD4^+^ T cells from healthy control persons in regard to GZMB expression. The CD4^+^ T cells were stimulated with histamine, the H2R agonist amthamine, the H4R agonist ST-1006 but not with IL-4 to reflect the atopic milieu of the individual patients as unaffected as possible. No regulation of GZMB release was observed in CD4^+^ T cells from healthy control persons in response to histamine or the H2R agonist (Fig. 7D). However, we detected an up-regulation of GZMB protein concentration in the supernatants from CD4^+^ T cells derived from AD patients in response to histamine (median 1.74-fold induction) (Fig. 7E).

To rule out the role of the H4R in the release of GZMB protein we performed ELISA experiments stimulating the H2R and H4R in parallel. Here we detected a significant up-regulation of GZMB protein secretion mediated via the H2R. Stimulating the H4R had no effect (Fig. 7F).

## Discussion

**Fig. 8:**
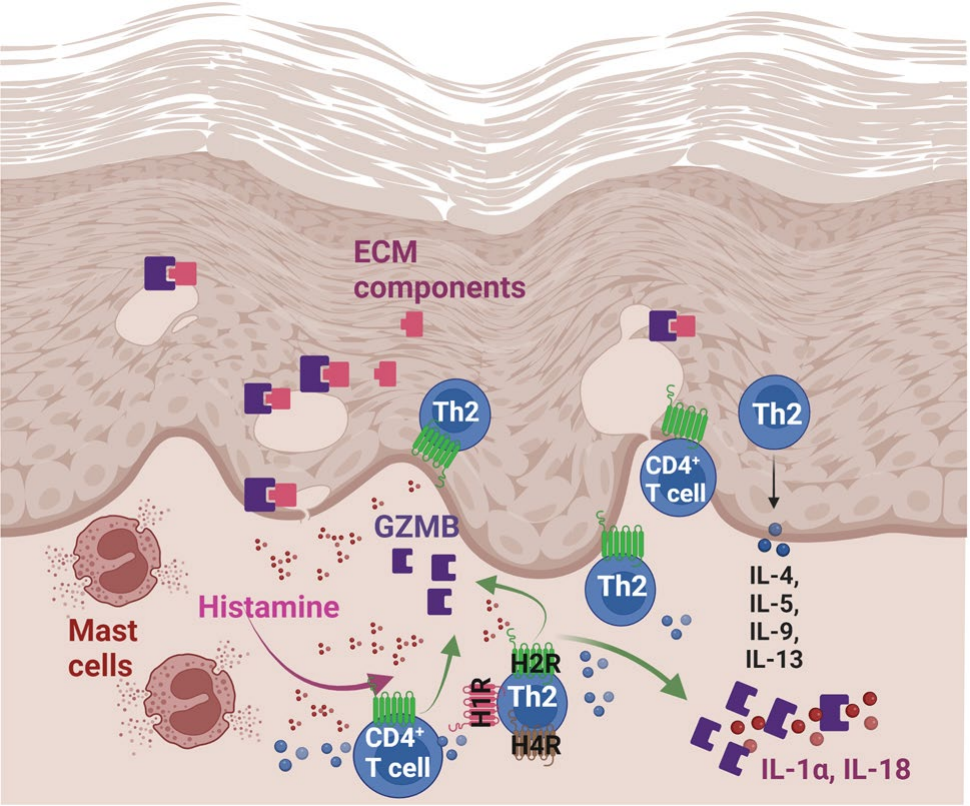
Histamine fosters the expression of granzyme B (GZMB) in CD4^+^ T cells in atopic dermatitis (schematic overview). In atopic dermatitis driven by Th2 cytokines, in particular by IL-4, histamine is released mainly from activated mast cells and up-regulates GZMB mRNA expression and secretion via H2R in CD4^+^ T cells or in Th2-polarized CD4^+^ T cells. Beyond the intracellular capacity of GZMB to initiate apoptosis, GZMB is also present extracellularly in the skin and processes or activates the cytokines IL-1α or IL-18. GZMB has also the ability to degrade several extracellular matrix (ECM) components. In this context, the elevated levels of GZMB induced by histamine may contribute to the disease pathology (created with BioRender.com)

In this study, we used RNA-Seq using Illumina NextSeq 550 sequencer to detect transcripts expressed in response to histamine, a molecule highly abundant in atopic skin [27]. The transcriptomes from Th2-polarized CD4^+^ T cells from different healthy donors treated with or without histamine during the whole polarization process were analysed. The soluble protein GZMB, which is stored in secretory lysosomes of cytotoxic T cells was one of the highly up-regulated genes in Th2-polarized CD4^+^ T cells treated with histamine (Fig. 8). To confirm this prominent RNA-Seq result, we isolated naïve CD4^+^ T cells, polarized the cells into Th2 cells and stimulated the cells with histamine or agonists for the H1R, H2R and H4R for different time points. We observed a significant up-regulation of GZMB mRNA expression by histamine and by stimulating the H2R, whereas GZMA and PRF1 mRNA expressions were not regulated by histamine although an increase of the number PRF1 transcripts were detected in RNA-Seq. The histamine-mediated secretion of various Th1 and Th2 cytokines from CD4^+^ T cells during the time of differentiation to Th2-polarized CD4^+^ T cells may cause indirect or reinforcing effects. These could explain the up-regulation of PRF1 in this long-term protocol.

In accordance to our data, Grossman et al. [28] recognized constitutive GZMB expression levels in resting CD4^+^ T cells. However, activation of these cells by ConA/IL-2 or by antibodies against the T cell receptor CD3 or against the co-stimulatory molecule CD28 induced higher levels of GZMB expression, but not of GZMA [28]. We detected high constitutive expression levels of GZMB in CD3/CD28 activated Th0-polarized CD4^+^ T or Th2-polarized CD4^+^ T cells when compared to non-activated total CD4^+^ T cells. However, Th2-polarized CD4^+^ T cells, which were stimulated with IL-4 during the polarization, showed lower constitutive GZMB expression in comparison to Th0-polarized CD4^+^ T cells.

Histamine and the H2R agonist were also able to induce GZMB protein production in Th2-polarized CD4^+^ T cells. Significantly higher amounts of spot forming colonies, representing the GZMB-secreting cells were detected in histamine-stimulated cells when compared to non-stimulated cells, and higher GZMB protein levels were measured in the supernatants of histamine and H2R agonist stimulated cells. The higher mRNA expression levels of the H2R when compared to expression levels of the H4R in Th2-polarized CD4^+^ T cells support a dominant role of the H2R in the histamine-mediated GZMB regulation.

Jutel et al. [18, 29] detected also predominant expression of the H2R mRNA in Th2-polarized CD4^+^ T cells when compared to Th1 polarized CD4^+^ T cells. Since the H4R was found in the year 2000 [30], its expression on T cells was not examined in their study. Gutzmer et al. showed that the H4R mRNA is higher expressed in Th2 cells when compared to Th1 cells [19].

To examine the cells in a more unaffected state, we isolated total CD4^+^ T cells and treated the cells for 72 h with IL-4 without co-stimulation. An up-regulation of GZMB mRNA expression in response to histamine or to an H2R agonist was also detectable in these cells. Importantly, we detected up to 600-fold higher constitutive GZMB mRNA expression in Th2-polarized CD4^+^ T cells when compared to CD4^+^ T cells treated only with IL-4 for 72 h or to untreated CD4^+^ T cells obtained from healthy donors. Presumably, these different expression levels could be attributed to the activation via the TCR or via the co-stimulatory molecule CD28 as well as to the stimulation with cytokines during the polarization of Th2 cells.

Interestingly, the degree of up-regulation of GZMB upon stimulation via the H2R was stronger in CD4^+^ T cells when compared to Th2-polarized CD4^+^ T cells (5.35-fold median versus 1.82). This is may be related to the high constitutive GZMB expression in Th2-polarized CD4^+^ T cells which leaves less room for further up-regulation. Higher numbers of PRF1 and GZMB positive cells were detected by immunohistochemistry in lesional skin of AD- and psoriasis patients when compared to non-lesional or healthy skin of respective patients. However, the number of immunoreactive cells was significantly lower in psoriatic skin when compared to AD skin [31].

To mimic a realistic approach of the atopic situations, CD4^+^ T cells isolated from AD patients and from healthy donors were investigated upon stimulation with histamine or different agonists only. Remarkably, unstimulated CD4^+^ T cells from AD patients and cells stimulated with histamine or with H2R agonists showed elevated levels of GZMB protein release when compared to respective cells from healthy donors. Stimulating the cells with the H4R agonist had no effect.

Going along with former observations in the literature, our results point to an important role of GZMB in AD. The lower constitutive GZMB expression in Th2-polarized CD4^+^ T cells, cultured in the presence of IL-4 suggests that GZMB is downregulated by this key cytokine of Th2 inflammation. This has previously been observed in CD8^+^ T cells where IL-4 suppressed the generation of the cytotoxic effector molecules granzyme A, B and C [32, 33].

In addition, the up-regulation of GZMB expression by histamine in Th2-polarized CD4^+^ T cells, in CD4^+^ cells activated by IL-4 or in CD4^+^ T cells from AD patients indicates that this mediator of allergic inflammation may play a role in the homeostasis of the expression of GMZB in atopy and allergy.

Beyond its direct effects, IL-4 appears to prime cells for responses to different stimuli, in particular to histamine. In accordance to our data, it was demonstrated that IL-4 primes human endothelial cells or human M2 macrophages for secondary responses by histamine showing up-regulation of PGE2 release [34] or enhanced production of M2 macrophage specific chemokines CCL17 and CCL18 in respective cells [35, 36].

Our study points to a novel mechanism of histamine mainly by stimulating CD4^+^ T cells via the H2R to accumulate GZMB extracellularly in the tissue and thereby triggering its diverse extracellular activities.

GZMB potently enhances the biological activity of cytokines in the tissue by converting the pro-form of IL-1α or of IL-18 into the authentic mature cytokine leading to prolonged inflammation [37]. Vitronectin, fibronectin, laminin, cadherin and filaggrin were identified as extracellular matrix substrates of GZMB where the protease cleaves these matrix proteins at a common binding site leading to disruption and cell detachment [10, 38]. In inflammatory conditions stimulating the H2R may thus contribute to an impaired epidermal barrier function, one major hallmark of AD, by increasing the levels of GZMB production. Blocking the H2R could be a therapeutic option, in this regard a recent study presents promising data about anti-inflammatory and anti-oxidant activities of the competitive histamine H2 receptor antagonist roxatidine acetate hydrochloride [39]. Apart from the regulation of GZMB, stimulating via the H2R exerts diverse functions on T cells. Histamine has shown to act as a stimulatory signal for the initiation of allergic inflammation by increasing IL-5 production in human Th2 cells [40], whereas Kunzmann et al. [41] showed that histamine interacts with TGF-β mediating the suppression of IL-4 production in human Th2 cells. An inhibition of IL-4 and IL-13 secretion was also observed by an H2R agonist in mice Th2-polarized CD4^+^ T cells which provides a negative signal for T cells [18].

In addition to our observations on human M2 macrophages [36] this study provides evidence that the H2R tends to show an inflammatory profile in allergic situations also on human CD4^+^ T cells.

## Supplementary Information

Below is the link to the electronic supplementary material.

Supplementary file1 Supplementary Fig. 1: Purity of the isolated naive CD4+ T cells. Naïve CD4+ T cells were isolated from PBMCs by magnetic labelling. The cells were fluorescently stained with anti-CD45RA-Phycoerythrin (PE) and anti-CD4-Allophycocyanin (APC) and analysed by flow cytometry (A) Forward-Scatter (FSC) and Side-Scatter (SSC). (B) Isotype control IgG2b-PE and IgG1-APC (C) Cells labelled with anti-CD45RA-PE and anti-CD4-APC. Percentages CD4+ and CD45RA+ T-cells are indicated in the upper right quadrant. Representative experiment out of 8 (JPG 126 KB)

Supplementary file2 Supplementary Fig. 2: Purity of the isolated CD4+ T cells. CD4+ T cells were isolated from PBMCs by magnetic labelling. The cells were stained with anti-CD3-Phycoerythrin (PE) and anti-CD4-Allophycocyanin (APC) and analysed by flow cytometry (A) Forward-Scatter (FSC) and Side-Scatter (SSC). (B) Isotype control IgG1-PE and IgG1-APC (C) Cells labelled with anti-CD3-PE and anti-CD4-APC. Percentages of CD4+ and CD3+ T-cells are indicated in the upper right quadrant. Representative experiment out of 11 (JPG 121 KB)

Supplementary file3 Supplementary Figure 3: The up-regulation of granzyme B (GZMB) mRNA expression in Th2-polarized CD4+ T cells is partly inhibited by pre-incubation the cells with the H2R antagonist ranitidine. Th2-polarized CD4+ cells were pre-incubated for 30 min A, with the H2R antagonist ranitidine before stimulation with the H2R agonist amthamine for 24 h, B, with the H4R antagonist JNJ7777120 before stimulation with the H4R agonist ST-1006 for 24 h, (concentration of each ligand 10 µM). GZMB expression was detected by q-PCR. GZMB mRNA expression relative to the rps 20 mRNA expression (reference gene) is shown as target/reference (GZMB/RPS20) ratio. P values were calculated by the Wilcoxon matched-pairs signed-rank test. Data are shown as individual values with medians. A and B (n = 5 independent donors and experiments) (JPG 159 KB)

## Data Availability

A data availability statement for this journal is provided by the authors.

## References

[1] Boivin WA, Cooper DM, Hiebert PR, Granville DJ (2009). Intracellular versus extracellular granzyme B in immunity and disease: Challenging the dogma. Lab Invest.

[2] Thornberry NA, Rano TA, Peterson EP, Rasper DM, Timkey T, Garcia-Calvo M (1997). A combinatorial approach defines specificities of members of the caspase family and granzyme B functional relationships established for key mediators of apoptosis. J Biol Chem..

[3] Tamang DL, Redelman D, Alves BN, Vollger L, Bethley C, Hudig D (2006). Induction of granzyme B and T cell cytotoxic capacity by IL-2 or IL-15 without antigens: multiclonal responses that are extremely lytic if triggered and short-lived after cytokine withdrawal. Cytokine.

[4] Griffiths GM, Isaaz S (1993). Granzymes A and B are targeted to the lytic granules of lymphocytes by the mannose-6-phosphate receptor. J Cell Biol.

[5] Adrain C, Murphy BM, Martin SJ (2005). Molecular ordering of the caspase activation cascade initiated by the cytotoxic T lymphocyte/natural killer (CTL/NK) protease granzyme B. J Biol Chem.

[6] Spaeny-Dekking EH, Kamp AM, Froelich CJ, Hack CE (2000). Extracellular granzyme A, complexed to proteoglycans, is protected against inactivation by protease inhibitors. Blood.

[7] Pardo J, Wallich R, Ebnet K, Iden S, Zentgraf H, Martin P (2007). Granzyme B is expressed in mouse mast cells in vivo and in vitro and causes delayed cell death independent of perforin. Cell Death Differ.

[8] Hendel A, Hsu I, Granville DJ (2014). Granzyme B releases vascular endothelial growth factor from extracellular matrix and induces vascular permeability. Lab Invest.

[9] Isaaz S, Baetz K, Olsen K, Podack E, Griffiths GM (1995). Serial killing by cytotoxic T lymphocytes: T cell receptor triggers degranulation, re-filling of the lytic granules and secretion of lytic proteins via a non-granule pathway. Eur J Immunol.

[10] Buzza MS, Zamurs L, Sun J, Bird CH, Smith AI, Trapani JA (2005). Extracellular matrix remodeling by human granzyme B via cleavage of vitronectin, fibronectin, and laminin. J Biol Chem.

[11] Berthou C, Michel L, Soulie A, Jean-Louis F, Flageul B, Dubertret L (1997). Acquisition of granzyme B and fas ligand proteins by human keratinocytes contributes to epidermal cell defense. J Immunol.

[12] Weidinger S, Beck LA, Bieber T, Kabashima K, Irvine AD (2018). Atopic dermatitis. Nat Rev Dis Primers.

[13] Werfel T, Allam JP, Biedermann T, Eyerich K, Gilles S, Guttman-Yassky E (2016). Cellular and molecular immunologic mechanisms in patients with atopic dermatitis. J Allergy Clin Immunol.

[14] Werfel T (2009). The role of leukocytes, keratinocytes, and allergen-specific IgE in the development of atopic dermatitis. J Invest Dermatol.

[15] Kamata Y, Kimura U, Matsuda H, Tengara S, Kamo A, Umehara Y (2016). Relationships among plasma granzyme B level, pruritus and dermatitis in patients with atopic dermatitis. J Dermatol Sci.

[16] Langan SM, Irvine AD, Weidinger S (2020). Atopic dermatitis. Lancet.

[17] Lang CCV, Renert-Yuval Y, Del Duca E, Pavel AB, Wu J, Zhang N (2021). Immune and barrier characterization of atopic dermatitis skin phenotype in Tanzanian patients. Ann Allergy Asthma Immunol.

[18] Jutel M, Watanabe T, Klunker S, Akdis M, Thomet OA, Malolepszy J (2001). Histamine regulates T-cell and antibody responses by differential expression of H1 and H2 receptors. Nature.

[19] Gutzmer R, Mommert S, Gschwandtner M, Zwingmann K, Stark H, Werfel T (2009). The histamine H4 receptor is functionally expressed on T(H)2 cells. J Allergy Clin Immunol.

[20] Hanifin JM, Rajka G (1980). Diagnostic features of atopic dermatitis. Acta Dermatovener (Stockholm).

[21] Sander K, Kottke T, Stark H (2008). Histamine H3 receptor antagonists go to clinics. Biol Pharm Bull.

[22] Gschwandtner M, Koether B, Werfel T, Stark H, Gutzmer R (2013). Profiling of histamine H4 receptor agonists in native human monocytes. Br J Pharmacol.

[23] Bustin SA, Benes V, Garson JA, Hellemans J, Huggett J, Kubista M (2009). The MIQE guidelines: minimum information for publication of quantitative real-time PCR experiments. Clin Chem.

[24] Bryceson YT, Fauriat C, Nunes JM, Wood SM, Björkström NK, Long EO (2010). Functional analysis of human NK cells by flow cytometry. Methods Mol Biol.

[25] Grinde B (1983). Effect of carboxylic ionophores on lysosomal protein degradation in rat hepatocytes. Exp Cell Res.

[26] Watanabe S, Yamada Y, Murakami H (2020). Expression of Th1/Th2 cell-related chemokine receptors on CD4(+) lymphocytes under physiological conditions. Int J Lab Hematol.

[27] Ruzicka T, Gluck S (1983). Cutaneous histamine levels and histamine releasability from the skin in atopic dermatitis and hyper-IgE-syndrome. Arch Dermatol Res.

[28] Grossman WJ, Verbsky JW, Tollefsen BL, Kemper C, Atkinson JP, Ley TJ (2004). Differential expression of granzymes A and B in human cytotoxic lymphocyte subsets and T regulatory cells. Blood.

[29] Jutel M, Klunker S, Akdis M, Malolepszy J, Thomet OA, Zak-Nejmark T (2001). Histamine upregulates Th1 and downregulates Th2 responses due to different patterns of surface histamine 1 and 2 receptor expression. Int Arch Allergy Immunol.

[30] Yamaura K, Oda M, Suwa E, Suzuki M, Sato H, Ueno K (2009). Expression of histamine H4 receptor in human epidermal tissues and attenuation of experimental pruritus using H4 receptor antagonist. J Toxicol Sci.

[31] Yawalkar N, Schmid S, Braathen LR, Pichler WJ (2001). Perforin and granzyme B may contribute to skin inflammation in atopic dermatitis and psoriasis. Br J Dermatol.

[32] Baschuk N, Utermöhlen O, Gugel R, Warnecke G, Karow U, Paulsen D (2007). Interleukin-4 impairs granzyme-mediated cytotoxicity of simian virus 40 large tumor antigen-specific CTL in BALB/c mice. Cancer Immunol Immunother.

[33] Kienzle N, Buttigieg K, Groves P, Kawula T, Kelso A (2002). A clonal culture system demonstrates that IL-4 induces a subpopulation of noncytolytic T cells with low CD8, perforin, and granzyme expression. J Immunol.

[34] Wierzbicki T, Iqbal SM, Cuvelier SL, Awong G, Tibbles LA, Patel KD (2003). IL-4 primes human endothelial cells for secondary responses to histamine. J Leukoc Biol.

[35] Mommert S, Schaper JT, Schaper-Gerhardt K, Gutzmer R, Werfel T (2021). Histamine increases Th2 cytokine-induced CCL18 expression in human M2 macrophages. Int J Mol Sci.

[36] Mommert S, Gregor K, Rossbach K, Schaper K, Witte T, Gutzmer R (2018). Histamine H2 receptor stimulation upregulates TH2 chemokine CCL17 production in human M2a macrophages. J Allergy Clin Immunol.

[37] Afonina IS, Tynan GA, Logue SE, Cullen SP, Bots M, Lüthi AU (2011). Granzyme B-dependent proteolysis acts as a switch to enhance the proinflammatory activity of IL-1α. Mol Cell.

[38] Turner CT, Zeglinski MR, Richardson KC, Zhao H, Shen Y, Papp A (2019). Granzyme K expressed by classically activated macrophages contributes to inflammation and impaired remodeling. J Invest Dermatol.

[39] Kang Y, Lee M, An H (2021). New potential of roxatidine acetate hydrochloride on atopic dermatitis mouse model, human keratinocytes, and human skin equivalent model. Front Pharmacol.

[40] Schmidt J, Fleissner S, Heimann-Weitschat I, Lindstaedt R, Szelenyi I (1994). Histamine increases anti-CD3 induced IL-5 production of TH2-type T cells via histamine H2-receptors. Agents Actions.

[41] Kunzmann S, Mantel PY, Wohlfahrt JG, Akdis M, Blaser K, Schmidt-Weber CB (2003). Histamine enhances TGF-beta1-mediated suppression of Th2 responses. FASEB J.

