# Supplementary figures and images for: The mRNA expression and secretion of granzyme B are up-regulated via the histamine H2 receptor in human CD4+ T cells
Source: Inflamm Res. 2023 Jul 20;72(8):1525–38. doi: 10.1007/s00011-023-01759-3 (PMC10499701; doi:10.1007/s00011-023-01759-3)

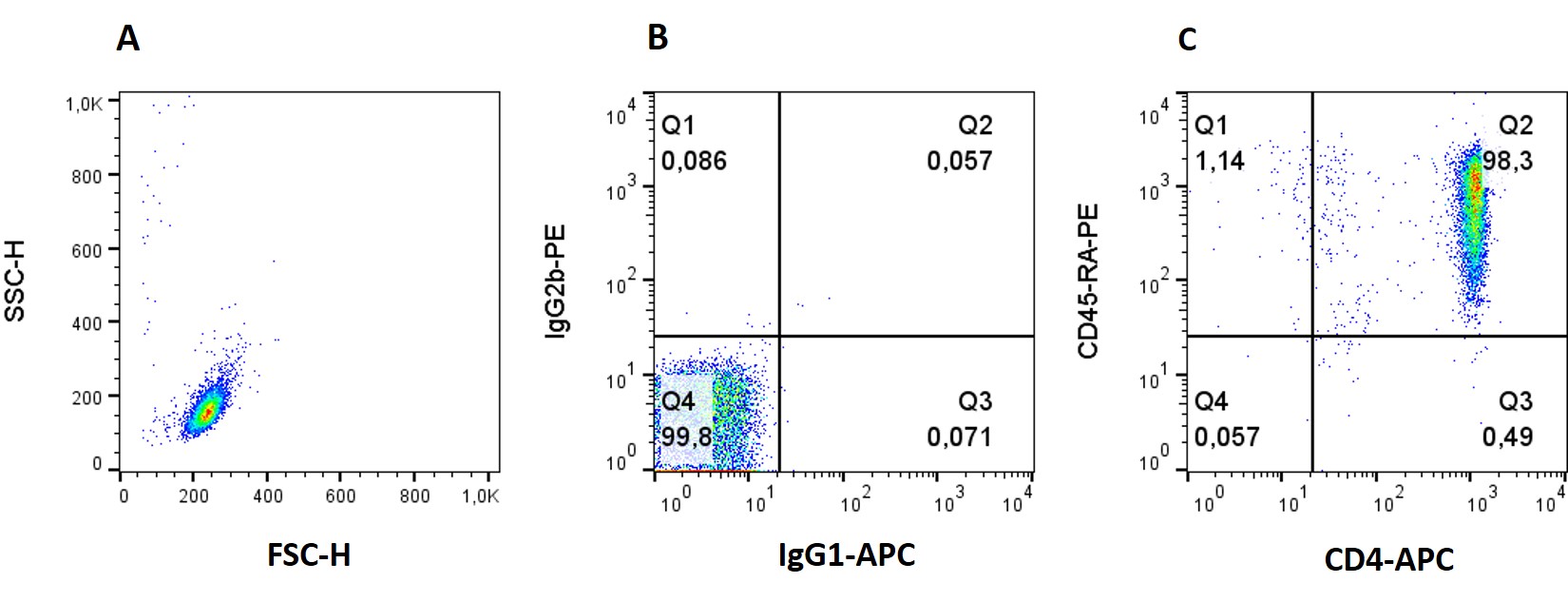

Supplement: Supplementary file 1 — Supplementary file1 Supplementary Fig. 1: Purity of the isolated naive CD4+ T cells. Naïve CD4+ T cells were isolated from PBMCs by magnetic labelling. The cells were fluorescently stained with anti-CD45RA-Phycoerythrin (PE) and anti-CD4-Allophycocyanin (APC) and analysed by flow cytometry (A) Forward-Scatter (FSC) and Side-Scatter (SSC). (B) Isotype control IgG2b-PE and IgG1-APC (C) Cells labelled with anti-CD45RA-PE and anti-CD4-APC. Percentages CD4+ and CD45RA+ T-cells are indicated in the upper right quadrant. Representative experiment out of 8 (JPG 126 KB) [file 11_2023_1759_MOESM1_ESM.jpg]

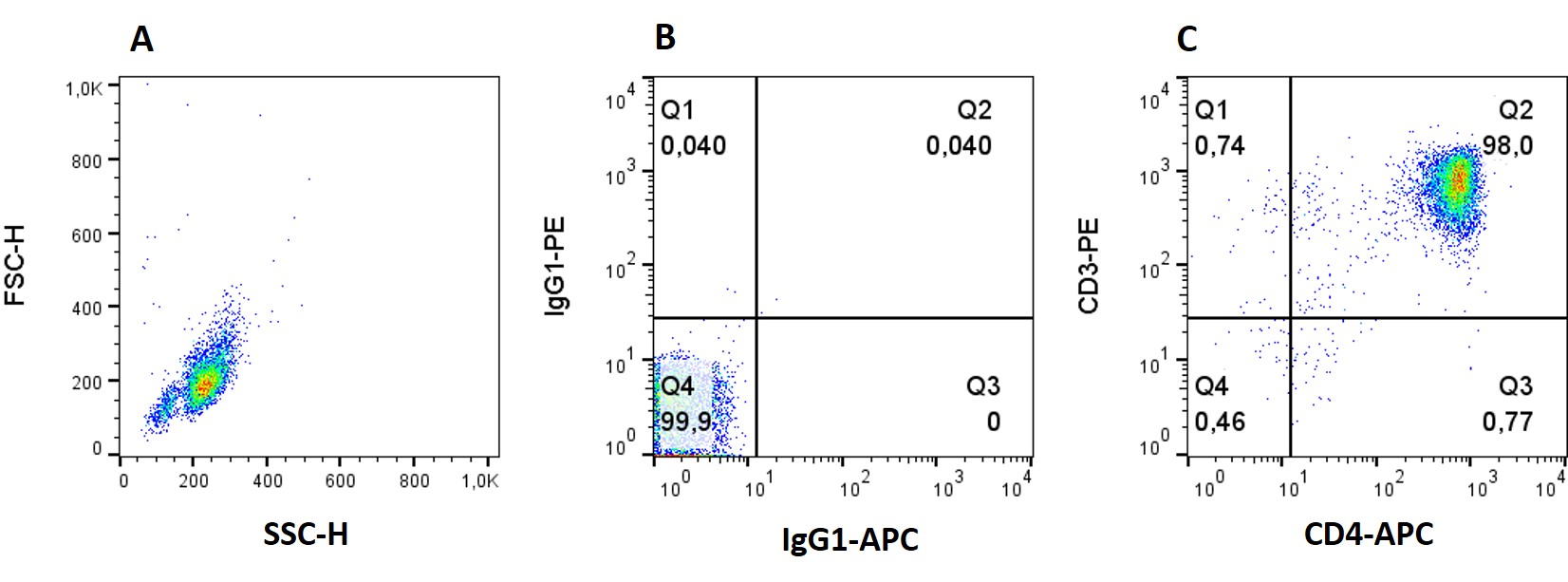

Supplement: Supplementary file 2 — Supplementary file2 Supplementary Fig. 2: Purity of the isolated CD4+ T cells. CD4+ T cells were isolated from PBMCs by magnetic labelling. The cells were stained with anti-CD3-Phycoerythrin (PE) and anti-CD4-Allophycocyanin (APC) and analysed by flow cytometry (A) Forward-Scatter (FSC) and Side-Scatter (SSC). (B) Isotype control IgG1-PE and IgG1-APC (C) Cells labelled with anti-CD3-PE and anti-CD4-APC. Percentages of CD4+ and CD3+ T-cells are indicated in the upper right quadrant. Representative experiment out of 11 (JPG 121 KB) [file 11_2023_1759_MOESM2_ESM.jpg]

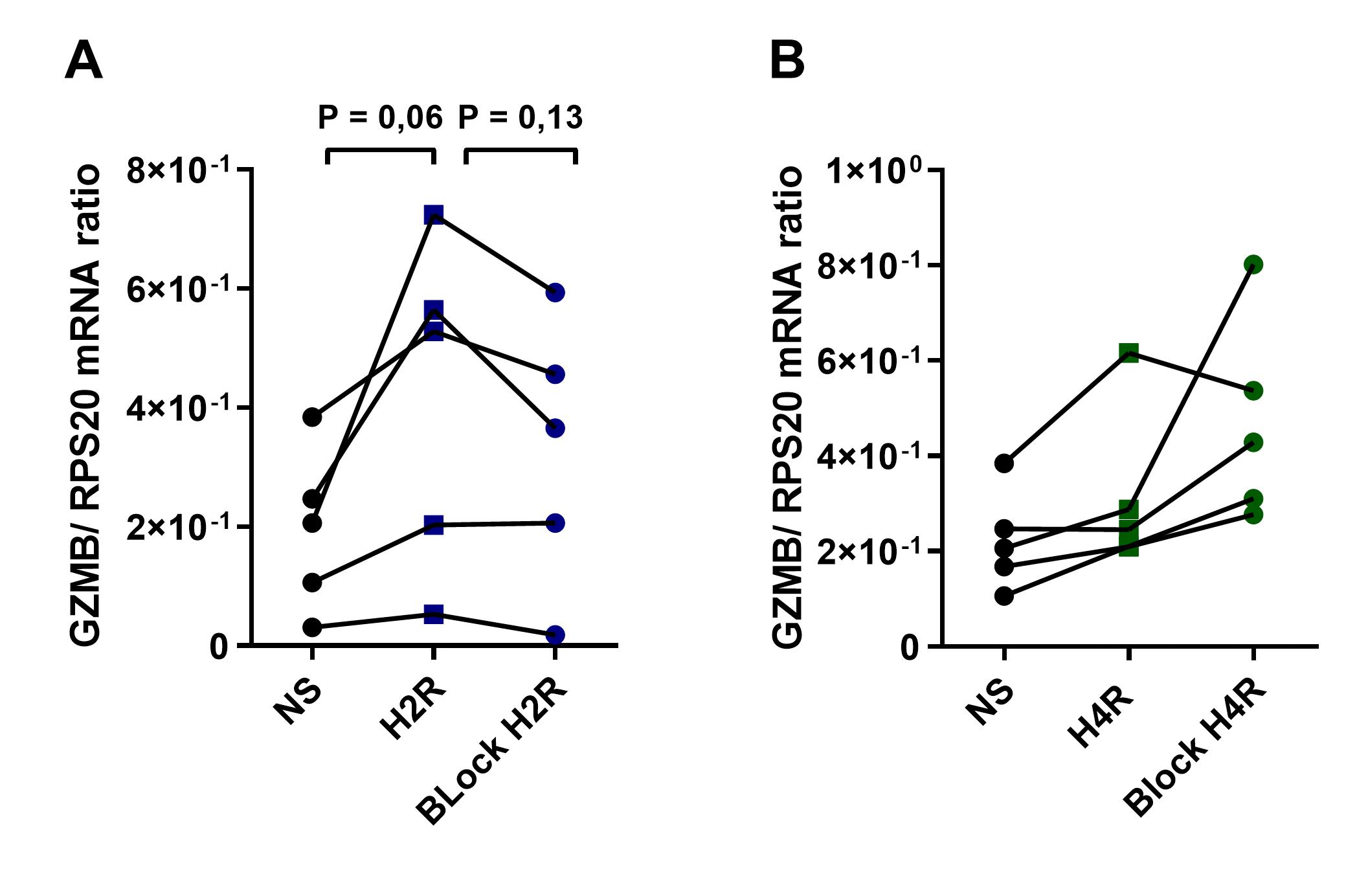

Supplement: Supplementary file 3 — Supplementary file3 Supplementary Figure 3: The up-regulation of granzyme B (GZMB) mRNA expression in Th2-polarized CD4+ T cells is partly inhibited by pre-incubation the cells with the H2R antagonist ranitidine. Th2-polarized CD4+ cells were pre-incubated for 30 min A, with the H2R antagonist ranitidine before stimulation with the H2R agonist amthamine for 24 h, B, with the H4R antagonist JNJ7777120 before stimulation with the H4R agonist ST-1006 for 24 h, (concentration of each ligand 10 µM). GZMB expression was detected by q-PCR. GZMB mRNA expression relative to the rps 20 mRNA expression (reference gene) is shown as target/reference (GZMB/RPS20) ratio. P values were calculated by the Wilcoxon matched-pairs signed-rank test. Data are shown as individual values with medians. A and B (n = 5 independent donors and experiments) (JPG 159 KB) [file 11_2023_1759_MOESM3_ESM.jpg]
